# Rumination as a Marker of Psychological Improvement in Transsexual Women Postoperative

**DOI:** 10.1089/trgh.2016.0029

**Published:** 2016-12-01

**Authors:** Andressa Mueller, Cláudia Quadros, Karine Schwarz, Angelo Brandelli Costa, Anna Martha Vaitses Fontanari, Bianca Machado Borba Soll, Dhiordan Cardoso da Silva, Maiko Abel Schneider, Érico de Moura Silveira, Marcia Kauer-Sant'Anna, Maria Inês Rodrigues Lobato

**Affiliations:** ^1^ Gender Identity Program (PROTIG), Hospital de Clinicas de Porto Alegre (HCPA), Universidade Federal do Rio Grande do Sul (UFRGS), Porto Alegre, Brazil.; ^2^ Pós Graduação em Psicologia, Pontifícia Universidade Católica do Rio Grande do Sul (PUC-RS), Porto Alegre, Brazil.; ^3^ Laboratory of Molecular Psychiatry, INCT for Translational Medicine, Hospital de Clínicas de Porto Alegre (HCPA), Universidade Federal do Rio Grande do Sul, Porto Alegre, Brazil.

**Keywords:** affirmation surgery, gender, gender dysphoria, gender transition, mental health, rumination

## Abstract

**Purpose:** This study aimed to analyze rumination levels of transsexual women before and after gender affirmation surgery (GAS). Rumination scores may represent a broader measure of GAS success and an alternative to patient-reported satisfaction, quality of life, well-being, or the presence of “caseness” for anxiety or depression as previously established in the literature.

**Methods:** Thirty-nine transsexual women were recruited. The participants completed the rumination scale of the Response Styles Questionnaire (RSQ) and were divided into three subsets according to the treatment time.

**Results:** The rumination scores were lower in the transsexual women who had undergone surgical procedures on primary sexual characteristics and gradually decreased with each additional procedure completed with respect to secondary sexual characteristics.

**Conclusion:** Rumination appears to comprise an important marker of improvement in post-GAS transsexual women.

## Introduction

Gender dysphoria (GD) (DSM5) refers to a striking incongruity between the experienced gender and the sex assigned at birth. This incongruity leads to discomfort with biological sexual characteristics and sometimes is followed by hormonal treatment and surgical interventions to make the body consistent with the desired gender. Recently, the Research Domain Criteria (RDoC) project created a novel program of research decoupled from classic etiological concerns, with a focus on investigating the biological and cognitive mechanisms and altered emotional states associated with mental health conditions.^1^ The investigation of basic cognitive processes, such as rumination as a transdiagnostic possibility, may therefore represent an important component of the RDoC program.^2^

Rumination as a factor related to psychopathology has been the subject of studies for at least three decades. The majority of these studies have been conducted with samples of depressed patients. The phenomenon of rumination is defined as involuntary and persistent self-centered thoughts,^3^ and it is associated with a constantly negative mood. Stress or adverse life events activate negative beliefs regarding oneself, which may manifest as rumination. Rumination, in turn, may act as a trigger for a depressive mood and, as a consequence, a depressive syndrome.^3^ The repetitive characteristics of ruminant thoughts set up a cycle in which patients focus on the causes and consequences of the symptoms of anxiety, which compromises their capacity to solve problems in an assertive manner and relieve themselves from the altered emotional state. Studies have demonstrated that active and adaptive coping strategies, which aim to visualize actions with the objective of solving problems, are negatively correlated with rumination.^4^

Rumination is a potential marker of mental diseases and emotional disorders. For example, evidence relates rumination to depression and episodes of major depression,^5^ symptoms of anxiety,^5^ social anxiety,^6^ generalized anxiety disorder,^5^ trauma, stress-related disorders, and suicidal ideation.^7^ Furthermore, high levels of rumination are associated with low self-esteem and predict excessive alcohol consumption, as well as symptoms of alcohol abuse in the long term.^8^ One factor that may increase rumination is chronic stress, that is, social and environmental circumstances that force an organism to physiologically and psychologically adapt over time.^9^ The process of stress involves a dynamic interaction between the environment and the organism, which changes over time in response to external challenges, perceptions of these challenges, and activated coping strategies.^9^

Gender affirmation surgery (GAS) is essential for relieving GD in individuals who desire to change their primary sexual characteristics to match their gender identity. Follow-up studies demonstrated undeniable beneficial effects from GAS, such as improvements in subjective well-being and sexual function.^10–12^ Additional benefits are related to social adaptation,^13^ for example, initiating and maintaining relationships are easier following GAS.^12^ However, 15 years after GAS, the quality of life has been verified to be lower in the domains of general health, role limitation, physical limitation, and personal limitation.^14^ Quality of life, regardless of performing surgery, is lower in transgender people when compared to cisgender persons.^15^

This study was designed to contribute to the literature by investigating markers of positive GAS outcomes, and the objective is to analyze rumination levels in transsexual women before and after GAS. Our rationale is that rumination scores may represent a broader measure of GAS success and an alternative to patient-reported satisfaction, quality of life, well-being, or the presence of “caseness” for anxiety or depression as previously established in the literature. Our hypothesis is that preoperative individuals will exhibit increased rumination levels compared with those who are receiving psychological counseling while awaiting GAS and compared with those who have undergone GAS.

## Methods

Participants were recruited from a GD outpatient clinic at Hospital de Clínicas de Porto Alegre (HCPA), a university hospital situated in southern Brazil. HCPA is the only public hospital currently performing GAS in the country, the only reference for specialized care for GD in southern Brazil and one of the primary Latin American centers for GD studies. Since its creation in 1998, the program for GD care has been conducting multidisciplinary outpatient treatment, which includes psychology, hormonal therapy, and surgical options to individuals diagnosed with GD.

The Ethics Committee of HCPA approved this study. All subjects were advised regarding the procedure and signed informed consent forms before study participation.

### Participants

The sample comprised 45 transsexual women, recruited during 2014 and 2015, who met the inclusion and exclusion criteria, respectively, having DSM5 criteria for GD and being free from cognitive impairment, psychosis, and/or dementia (assessed by a psychiatric interviewer). All patients from a GD outpatient clinic, after a well-established diagnosis, were personally invited to join the study, thereby composing a consecutive sample. None of the participants received financial support. Five volunteers did not complete data collection and one did not achieve a minimum IQ score, which resulted in a final sample of 39 individuals.

This sample was divided into three subsets on the basis of different stages of treatment. The first subset comprised 13 participants at the start of treatment (T0), that is, they had previously been assessed by the team, had a confirmed diagnosis of GD, and had been receiving counseling for not >1 year. The second subset (T1) comprised 14 patients attending group counseling between 1 and 2 years and had no contraindications for GAS. The third subset (T2) contained 12 patients who had undergone GAS for a minimum of 6 months earlier.

### Measures

A semistructured interview (self-report) was conducted to obtain information regarding psychological and emotional development and medical history, which identified psychological suffering during childhood and adolescence and health-related vulnerabilities.

A version of the rumination scale of the Response Styles Questionnaire (RSQ) (Nolen-Hoeksema, 1991, revised by Treynor et al.^16^), adapted and translated into Portuguese, was used to identify factors of ruminative processes among the transsexual participants; this version only measured the rumination factor and comprised a 10-item scale.

Current psychiatric diseases were evaluated by the Mini International Neuropsychiatric Interview (MINI).^17^ The MINI has been validated in Brazil with adequate validity and reliability.^18^ The purpose of using MINI was only for screening for psychopathologies associated with rumination, such as depression and anxiety, in the three analyzed subsets.

The Brazilian version of the Wechsler Intelligence Scale for adults^19^ was used to assess intelligence to identify protective factors that may be related to rumination. Only two of the subtests were administered: vocabulary and cubes. The MINI test and the intelligence test were both used to exclude subjects who had a diagnosis of psychosis and/or an IQ <80.

The Portuguese translation^20^ of the Wagnild and Young Resilience Scale was implemented to investigate protective factors related to rumination.

### Statistical analysis

Analyses were performed using Statistical Product and Service Solutions (SPSS), version 18.0. The mean scores for rumination were compared across the three subsets using an analysis of variance. *T*-tests for independent samples were applied to identify differences between means in the main variables. Chi-square tests were used to calculate the psychiatric morbidities between the groups. Descriptive analyses were used to identify the most significant results from the structured interviews. Medical gender-affirmation procedures were operationalized as follows: the participants who had not undergone any type of surgical procedure on primary sexual characteristics scored (0), whereas a score of (1) was assigned to the individuals who had undergone an intervention to change primary sexual characteristics. The persons who had undergone procedures to change primary sexual characteristics scored an additional point (2) for procedures on secondary sexual characteristics, such as mammoplasty, facial feminization surgery, and thyroid cartilage reduction.

## Results

The majority of the sample (*n*=38) comprised residents of southern Brazil, the states of Rio Grande do Sul, Santa Catarina, and Paraná. Only one participant originated from the Mid-West of Brazil. The median age of the interviewees was 30 years with an interquartile range of 26–37. The mean number of years spent in education was 12.05 years with a standard deviation of 3.308. According to self-report, our sample was distributed by race/ethnic groups as follows: 71.79% white, 5.13% black, 17.95% *pardo* (black and white mixed race), and 5.13% multiracial. There were no indigenous individuals in the sample.

Table 1 indicates the distributions of the variables in the sample groups. The psychiatric assessment (MINI) to measure Axis I disorders at the time of assessment indicated that 56.41% of the participants were negative for any diagnosis, whereas 17.95%, 15.38%, and 10.26% were positive for at least one, two, and more than two diagnoses, respectively. The psychiatric assessment indicated that depression and generalized anxiety, in this order, comprised the diagnoses with the highest rates.

**Table 1.** **Sample Characteristics by Group**

	T0, *n* (13)	T1, *n* (14)	T2, *n* (12)
Rumination, ẋ±SD	23.77±6.954	20.43±3.777	17.33±4.677
GAS, *n* (%)	—	—	12 (100)
Procedures on secondary sexual characteristics, *n* (%)	—	1 (7.1)	9 (75)
Age, median [IQR]	26 [24.5/32.5]	30.50 [25.75/36]	32.5 [29.25/46.25]
Intelligence, ẋ±SD	100.38±11.63	106.46±11.84	100.83±10.63
Resilience, median [IQR]	143 [133–150]	147 [131.5–151.75]	157.5 [120–159]
Psychiatric morbidity, *n* (%)	7 (53.85)	6 (42.86)	4 (33.33)

GAS, Gender affirmation surgery; IQR, interquartile range; SD, standard deviation.

**FIG. 1.**
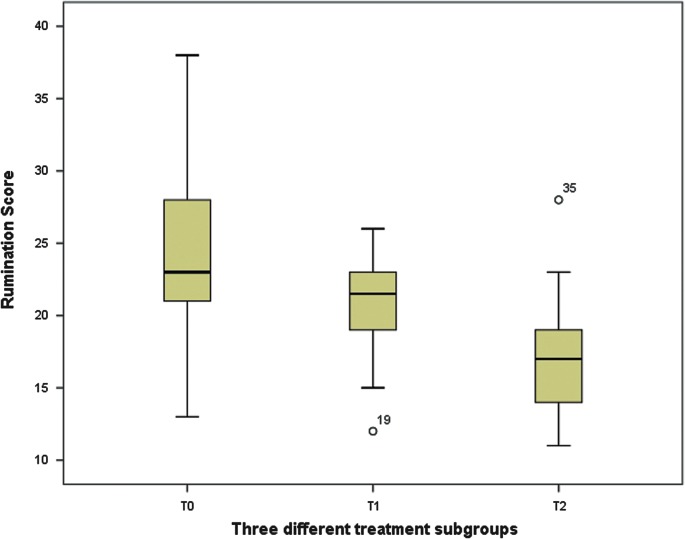
Rumination and procedure on secondary sexual characteristics undergone by the patients.

There were no significant differences between groups T0, T1, and T2 in terms of mean intelligence [*F*(2, 38)=1.72, *p*=0.19], resilience [*F*(2, 38)=0.49, *p*=0.62], or psychiatric morbidities [*χ*^2^(2, 39)=1.07, *p*=0.58] (Table 1). Our primary finding indicates the differences in the mean rumination scores linked to the surgical procedures to change the primary sexual characteristics group [*F*(2, 38)=3.46, *p*<0.05, *η*^2^=0.16]. The rumination scores were lower in the patients who had undergone surgical procedures on primary sexual characteristics (GAS), and the rumination scores gradually further decreased for each procedure on secondary sexual characteristics undergone by the patients in this group (Fig. 1). When controlled in a linear regression, using the enter method, psychiatric morbidity was not statistically significant, which indicates it did not predict differences in the rumination scores (Table 2).

**Table 2.** **Variables Predictive of Rumination**

	Step 1			Step 2		
	Constant	GAS	Psychiatric morbidities	Constant	GAS and procedures on secondary sexual characteristics	Psychiatric morbidities
*B*	18.87	−3.82	1.61	19.30	−2.47	1.54
*SE B*	1.90	1.86	0.82	1.85	0.96	0.80
*β*	*—*	0.31^**^	0.29	—	−0.38^*^	0.28

Enter method. *R*^2^=0.23 for step 1, *R*^2^=0.27 for step 2.

^*^ *p*<0.01, ^**^p<0.05.

## Discussion

As the transsexual women engaged in our study achieved physical characteristics that were more consistent with the gender they desired, they enjoyed benefits in terms of psychological adaptation, which manifested as a reduction in rumination scores. Nevertheless, it remains necessary to better understand this psychological adaptation: is it a result of alleviating GD or receiving more social acceptance?

We believe that there is a relationship between these two factors: GAS could make individuals more confident and thereby indirectly improve their physical appearance. This concept is congruent with the psychobiological theory of depression that states stressful experiences, particularly interpersonal stressors and social rejection, lead to rumination.^21^

Corroboration of the postulate that early body-gender misalignment could increase ruminant thoughts expressed as maladaptive mechanisms for the resolution of intrapsychic conflicts highlights the importance of considering early treatment in cases in which the diagnostic criteria for GD are present, particularly hormone suppression during preadolescence. There is evidence that transsexual persons have feelings of anxiety and depression at young ages, at ∼3–5 years old, which potentializes cognitive development in a nonadaptive manner.^22–24^ Psychiatric morbidities could be present from very early on in individuals with transsexuality, which emphasizes the need for early mental health promotion.

Early interventions with cognitive behavioral therapy could lead to the identification of ruminant thoughts and focus on changing patterns before the chronic and constant stress becomes a potential source of psychopathological internalization.^24^ This is particularly important in Brazil, where violence against transgender people is a constant source of chronic stress.^25^ In our study, adults in the pre-GAS group who were receiving counseling did not exhibit lower rumination scores when compared to patients who had undergone GAS. Due to the cross-sectional nature of our study, this finding does not present causality.

The cross-sectional nature and small sample size of our study are an important limitation. Although we proposed that performing GAS reduces rumination scores, it is possible that having lower rumination scores enabled some patients to remain attached to the clinic for at least 2 years before surgery. Therefore, a cross-sectional study cannot state that the stage of treatment helps to reduce rumination or whether the alternative is true, that is, the presence of lower levels of rumination (and likely lower levels of psychopathology) make it more likely that individuals progress to have surgery. All patients were assessed by a psychiatric interview following DSM parameters to be eligible for GAS, and we controlled for psychiatric morbidity in our regression analysis; thus, this design bias is minimized but not absent.

Another limitation of this study is the possibility of beta error with relation to protective mechanisms, such as intelligence and resilience, which may not have attained significance because of the small number of participants. Age was controlled in the rumination outcome; however, we consider a discussion of this variable to be indispensable.

It is expected that the current findings contribute to research within the RDoC paradigm, which aims to demonstrate relationships between psychological markers and mental health conditions. Rumination appears to comprise a marker of positive outcomes in post-GAS transsexual women.

## Abbreviations Used

GAS: Gender affirmation surgery
GD: gender dysphoria
HCPA: Hospital de Clínicas de Porto Alegre
RSQ: Response Styles Questionnaire
RDoC: Research Domain Criteria
SD: standard deviation
